# Health professional and transplant recipient perspectives of kidney transplantation in regional, rural, and remote Australia – a survey study

**DOI:** 10.1007/s40620-025-02331-4

**Published:** 2025-06-16

**Authors:** Tara K. Watters, Nicole J. Scholes-Robertson, Beverley D. Glass, Andrew J. Mallett

**Affiliations:** 1 College of Medicine & Dentistry, James Cook University, Townsville, QLD Australia; 2 Department of Renal Medicine, Cairns Hospital, PO Box 902, Cairns, QLD 4870 Australia; 3 Sydney School of Public Health, The University of Sydney, Sydney, NSW Australia; 4 Department of Renal Medicine, Townsville University Hospital, Townsville, QLD Australia; 5 Institute for Molecular Bioscience, The University of Queensland, Brisbane, QLD Australia

**Keywords:** Rural and remote health, Chronic kidney disease, Kidney transplant, Indigenous health

## Abstract

**Background:**

Despite higher rates of chronic kidney disease and kidney failure in rural and remote populations, these patients are less likely to receive a kidney transplant. Additional barriers to kidney transplantation are associated with the need to travel to metropolitan areas where medical testing and transplantation facilities are located. We determined the opinions, attitudes, and experiences of both health professionals and recent kidney transplant recipients regarding kidney transplantation processes in Australia for patients residing in regional, rural, and remote areas.

**Methods:**

A cross-sectional survey containing closed and open-ended questions was administered, with kidney transplant recipients from northern Queensland and Australian kidney transplant health professionals surveyed. Quantitative data were analysed using descriptive and inferential statistics, while a descriptive thematic method was used to analyse qualitative data.

**Results:**

Australian transplant health professionals (82) and recent kidney transplant recipients (77) participated. Almost all (97%) health professional participants agreed that receiving psychosocial support from peers would be beneficial for potential transplant recipients. Kidney transplant recipients rated information prior to transplant, potential medication side effects, high financial costs incurred related to treatment, and access to ongoing medication supply as the most important aspects in relation to their own experience. Prevalent themes around improving transplant experiences included enabling timely and flexible access to transplant assessment, reducing financial hardship, and fostering comprehensive psychosocial support.

**Conclusions:**

Multiple aspects of current kidney transplant processes in Australia, and particularly northern Australia, could be optimised to improve patient experiences and clinical outcomes for regional, rural, and remote kidney transplant recipients.

**Graphical Abstract:**

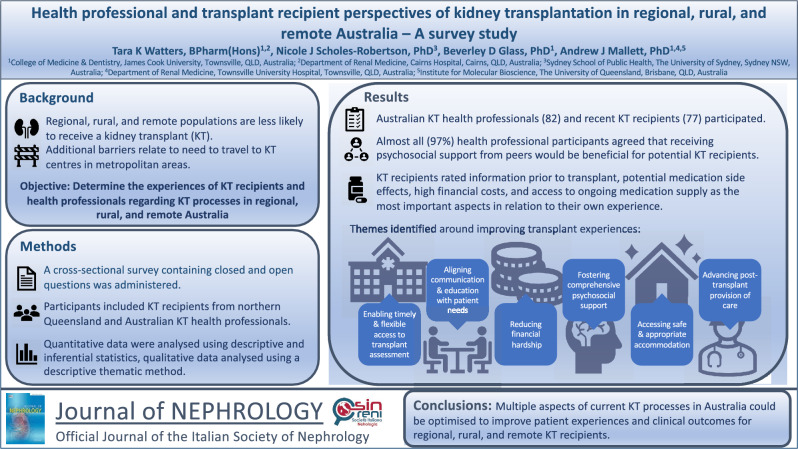

**Supplementary Information:**

The online version contains supplementary material available at 10.1007/s40620-025-02331-4.

## Introduction

An estimated 7 million people, or 29% of the total population of Australia reside in regional, rural, and remote areas [1], with these populations facing unique challenges when managing their health and wellbeing, often resulting in poorer health outcomes than their metropolitan counterparts [1]. Rural and remote populations globally face higher rates of chronic kidney disease (CKD) and kidney failure, and mortality rates almost twice as high compared to those patients in urban areas [2–4]. Outcomes for Indigenous populations worldwide are worse again, as they are twice as likely to progress to kidney failure than non-Indigenous patients, a risk that is compounded by residing in a rural or remote area [5, 6].

Despite the increased burden of CKD in rural and remote populations across the world, they are less likely to access specialist kidney health services, receive appropriate screening and education around CKD and kidney replacement therapy options, or receive a kidney transplant (KT) [7–9]. Many additional barriers to KT exist for this population, primarily associated with the need to travel in order to access medical testing and transplantation facilities [10]. Indigenous populations worldwide are even less likely to be deemed eligible for transplant, and experience significantly longer delays to KT waitlist activation [5, 11]. Recent data show that the overall number of Indigenous Australian KT recipients remains very low, at only 16% of those with treated kidney failure compared to 48% in the non-Indigenous population [12]. This is despite the proven survival benefit of KT when compared with remaining on dialysis [13].

There is research investigating access to all kidney replacement therapy modalities in rural and remote patient populations [14, 15], and research which has focused on attaining equity and removing cultural bias for Indigenous or minority populations accessing KT [16, 17]. However, there is still a paucity of data around improving access to KT for regional, rural, and remote populations, especially in northern Australia. The objective of this study was to determine the opinions, attitudes, and experiences of both health professionals and recent transplant recipients regarding KT processes for patients residing in regional, rural, and remote areas of Australia. The focus was on identifying ways in which access to transplantation can be improved, as well as improving the experiences and clinical outcomes for KT recipients from regional, rural, and remote areas.

## Methods

### Study design and recruitment

A survey was conducted across two separate participant groups. The first participant group consisted of Australian KT health professionals, including nephrologists, transplant nurses, clinical pharmacists, and social workers/Indigenous liaison officers. The second participant group included recent KT recipients from regional, rural, or remote areas of northern Queensland. Sampling was via a purposive non-probability method to appropriately answer the research objective. Geographical remoteness of participants was defined according to the Modified Monash Model (MMM) 2019 using residency and principal place of practice data [18]. Consent to participate was implied through survey completion. Further information regarding recruitment of both participant groups is included in Online Resource 1.

### Data collection

A structured cross-sectional survey containing closed questions (using both multiple choice and Likert scale response formats) plus one open-ended question, was designed specifically for each group of participants. Survey tools (Online Resource 2) were informed by a scoping review [10]. The Health Belief Model was also utilised as a framework to inform the design of the survey tool for KT recipients given the study investigated individual beliefs about a specific health condition that may influence individual health-related behaviours [19]. The survey tools were reviewed by all members of the research team to ensure validity of the content. Piloting was conducted with a sample cohort of four health professionals from different disciplines and two KT recipients that were not eligible to participate in the study, to ensure content clarity and readability before surveys were disseminated. Further information regarding data collection is included in Online Resource 1.

### Data analysis

Statistical analysis was conducted using SPSS (SPSS, version 29, IBM, Armonk United States). Categorical variables were described using the proportions and percentages of responses in each category. Frequency distributions of categorical variables were compared using chi-squared or Fisher’s exact tests [20]. Responses from 5-point Likert scale response questions were analysed as ordinal data and combined where appropriate (e.g., combining “strongly agree” and “agree”) to achieve adequate numbers for analysis.

Qualitative data from the open-ended survey question were analysed using a descriptive thematic method following the Braun and Clarke framework [21]. One investigator reviewed and coded the data, with a second investigator independently reviewing sections of data to confirm interpretation and reduce bias [22]. Further information regarding data analysis is included in Online Resource 1.

### Ethics approval

Multisite ethical approval was granted by the Townsville Hospital and Health Service Human Research Ethics Committee (HREC/2023/QTHS/89342).

## Results

### Health professionals

#### Characteristics of survey participants

Of the 82 responses received from eligible health professionals, participants included nephrologists from transplanting centres (18%), nephrologists from regional, rural, or remote non-transplanting centres (18%), clinical pharmacists (20%), nursing staff (24%), and other health professionals (20%). Over half of the participants were based in regional, rural, or remote areas (56%) and had > 7 years of experience in their profession (55%). Detailed demographic characteristics are presented in Table 1.

**Table 1:** Demographic characteristics of health professional participants

Health Professional Participant Characteristics	Value (*n* = 82)
Profession	
Nephrologist (transplanting)	15 (18%)
Nephrologist (non-transplanting)	15 (18%)
Clinical pharmacist	16 (20%)
Nursing staff	20 (24%)
Other	16 (20%)
Social worker or ILO	6
Dietitian	5
Psychologist	1
Transplant surgeon	1
Research coordinator	1
Registrar or advanced trainee	1
Medical officer at non-transplanting urban centre	1
Years of experience	
0–7 years	37 (45%)
7–14 years	21 (26%)
> 14 years	24 (29%)
Rurality of principal place of practice (MMM 2019)	
Metropolitan Area	36 (44%)
Regional Centre	34 (41%)
Large Rural Town	5 (6%)
Remote Community	4 (5%)
Very Remote Community	3 (4%)
Provision of transplant care^a^	
Pre-transplant	71 (87%)
Peri-transplant	34 (42%)
Post-transplant	70 (85%)
Modality used for provision of care^a^	
Face-to-face	81 (99%)
Telephone	62 (76%)
Telehealth pre-transplant	34 (42%)
Telehealth post-transplant	36 (44%)

^a^Some participants may have selected multiple options here

#### Health professional experiences

**Fig. 1:**
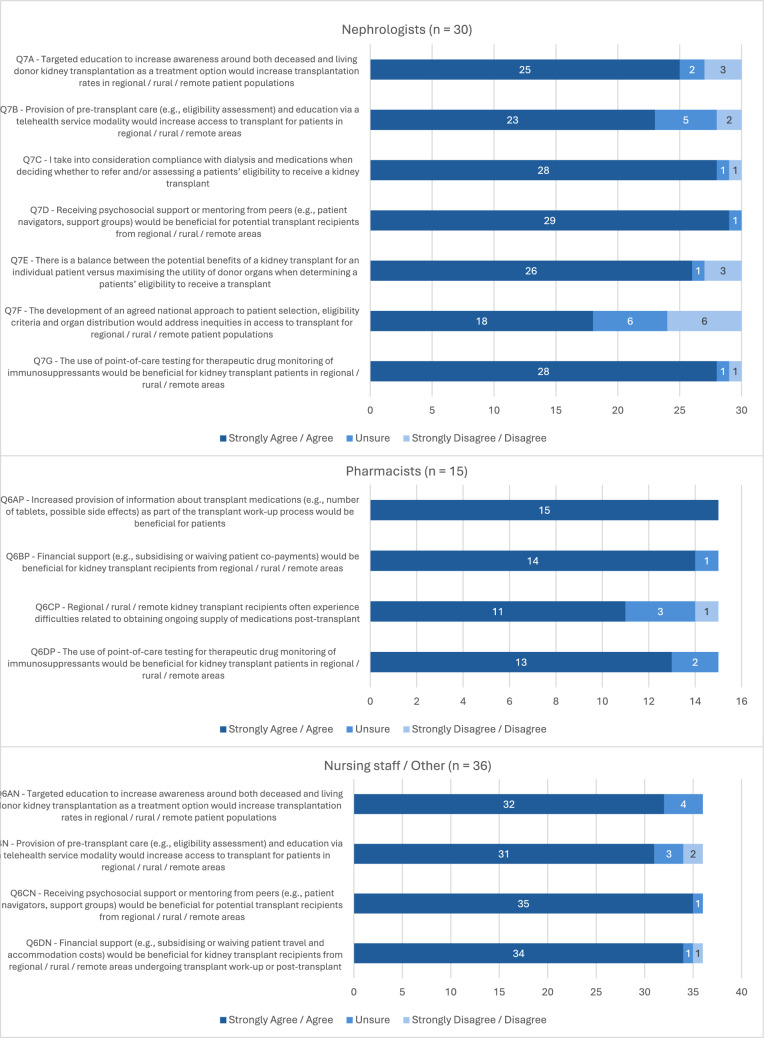
Health professional opinions around improving kidney transplant processes for regional, rural, and remote transplant recipients. Participant responses to Likert scale survey questions (*n* = 81)

Nephrologists (98%) reported discussing living donor kidney transplantation as an option with all potential KT recipients. Figure 1 provides a summary of participant Likert scale responses. Most nephrologists (83%) felt that targeted education to increase awareness around KT as a treatment option would increase transplantation rates in regional, rural, and remote patient populations. However, nephrologists working in transplant centres were more likely to agree with this statement compared to those in non-transplanting centres (*p* = 0.04).

Nephrologists (97%) and nursing staff/other health professionals (97%) agreed that receiving support or mentoring from other KT recipients would be beneficial for potential recipients. Over half of the nephrologists (60%) agreed that there is a balance between considering the potential benefits of a transplant for an individual patient, versus maximising the utility of donor organs when determining a patient’s eligibility to receive a transplant. However, nephrologists with > 7 years’ experience were more likely to agree with this statement compared to those with < 7 years’ experience (*p* = 0.01).

All pharmacists agreed that increased provision of medication information prior to transplant would be beneficial for potential recipients. Most pharmacists (73%) also felt that regional, rural, and remote KT recipients often experience difficulties obtaining ongoing supply of medications post-transplant. However, pharmacists based in regional, rural, or remote centres were more likely to agree with this statement compared to those based in metropolitan areas (*p* = 0.01).

When comparing the Likert responses to questions asked across both nephrologists and nursing staff/other health professionals participant groups, years of experience (*p* = 0.006) and profession (*p* = 0.02) were found to have a significant effect on agreement. Nephrologists and nursing staff/other health professionals with < 7 years’ experience were more likely to provide an ‘agree’ response than those with > 7 years’ experience (OR 0.22; 95% CI 0.08, 0.61). As a profession, nursing staff were more likely to provide an ‘agree’ response than nephrologists and other health professionals (OR 0.19; 95% CI 0.05, 0.76). Online Resource 3 includes full SPSS analysis output.

### Kidney transplant recipients

#### Characteristics of survey participants

A total of 77 responses were received from the 120 transplant recipients eligible to participate, achieving a response rate of 64%. Most participants received a deceased donor KT (82%) and had been on dialysis for > 1 year at the time of receiving their transplant (78%). Almost a third of participants were residing in rural or remote areas prior to transplant (30%), and a quarter identified as Indigenous Australians (25%). Detailed demographic characteristics are presented in Table 2.

**Table 2:** Demographic characteristics of kidney transplant recipient participants

Kidney transplant recipient participant characteristics	Value (*n* = 77)
Type of kidney transplant received	
Deceased donor	63 (82%)
Living related donor	5 (6.5%)
Living unrelated donor	4 (5%)
Unsure	5 (6.5%)
Years on dialysis prior to transplant	
Not on dialysis at time of transplant	4 (5%)
0–1 year	13 (17%)
1–3 years	33 (43%)
3–5 years	19 (25%)
> 5 years	8 (10%)
Rurality of residence at time of transplant (MMM 2019)	
Metropolitan Area	1 (1%)
Regional Centre	53 (69%)
Medium Rural Town	6 (8%)
Small Rural Town	4 (5%)
Remote Community	6 (8%)
Very Remote Community	7 (9%)
Aboriginal and/or Torres Strait Islander	
Aboriginal	11 (14%)
Torres Strait Islander	2 (3%)
Both	6 (8%)
Neither	54 (70%)
Prefer not to say	4 (5%)

#### Kidney transplant recipient experiences

The option of receiving a transplant from a living donor was discussed with only 73% of participants, and Indigenous Australians were less likely to have living donor KT discussed with them compared to non-Indigenous participants (*p* = 0.02). When asked to list in order of importance from 1 to 10, participants most frequently rated information prior to transplant, potential medication side effects, high financial costs incurred related to treatment, and access to ongoing medication supply as the most important aspects in relation to their own personal KT experience (Online Resource 4—Supplementary Fig. 1).

**Fig. 2:**
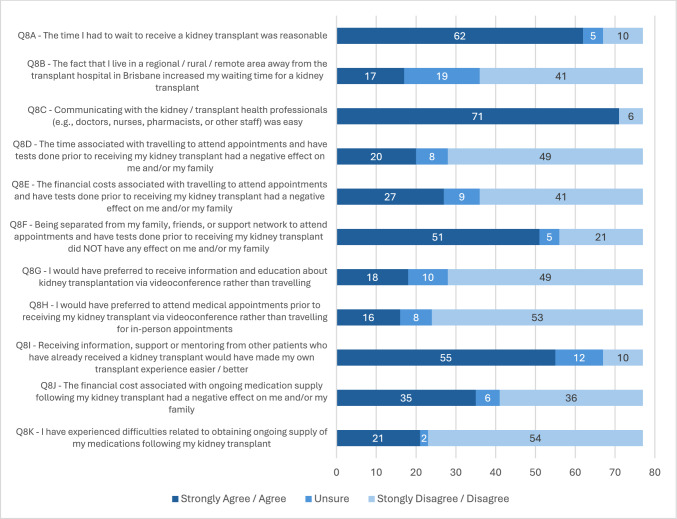
Experiences of recent kidney transplant recipients. Participant responses to Likert scale questions around participants’ own kidney transplant experience and potential ways in which their experience could have been improved (*n* = 77)

Figure 2 provides a summary of participant Likert scale responses. Most participants (81%) felt that the time they waited to receive a transplant was reasonable, however those who had been on dialysis for > 3 years at time of transplantation were less likely to agree with this compared to those on dialysis for < 3 years (*p* ≤ 0.001). There was a trend in the data suggesting an association between Indigenous status and a longer time spent on dialysis prior to transplant, however this was of borderline statistical significance (*p* = 0.05). Indigenous Australians were more likely to agree that residing further away from the transplant centre increased their wait time to transplant compared to non-Indigenous participants (*p* = 0.01). Indigenous participants were also more likely to be residing in a rural or remote area (*p* ≤ 0.001).

With regard to time spent away from home to complete work-up testing (prior to transplant) having a negative effect on patients and/or their families, the proportion of ‘agree’ responses was similar across both Indigenous and non-Indigenous participants. However, non-Indigenous participants were more likely to disagree with this statement compared to Indigenous participants (*p* = 0.01). The effect of rurality was similar, with metropolitan and regional participants more likely to disagree with this statement compared to rural and remote participants (*p* = 0.01).

Only 35% of participants agreed that the financial costs associated with travelling to complete work-up testing prior to transplant had a negative effect on them or their family. A trend in the data suggests that living donor KT participants were more likely to agree with this compared to those who received a deceased donor transplant, however this did not achieve statistical significance (*p* = 0.06).

There was an overall negative response from participants regarding the suggestion of telehealth for delivery of education and provision of medical appointments, with most reporting they would *not* prefer this option over travelling to attend in-person education (64%) or medical appointments (69%). Most participants (71%) agreed that receiving information and support from someone with lived experience of KT would have been beneficial. Almost half of the participants (45%) reported that the financial cost of ongoing medication supply was burdensome, and concerningly over a quarter (27%) reported experiencing difficulties accessing ongoing supply of their medications post-transplant.

When responses to the Likert scale questions were compared, degree of rurality of participants (*p* = 0.02) had a significant effect on agreement. Participants residing in regional or metropolitan areas were more likely to provide an ‘agree’ response compared to those residing in rural and remote areas (OR 0.67; 95% CI 0.49, 0.92), suggesting an overall more positive transplant experience for the metropolitan and regional participants. Online Resource 3 includes full SPSS analysis output.

### Qualitative responses

An open-ended question was posed at the end of the survey, which was consistent across both participant groups. This asked them to suggest a particular aspect of current KT processes (or their own transplant experience) that they would change to improve experiences and outcomes for regional, rural, and remote KT patients. Participant comments were thematically analysed together, and the identified themes and associated quotes are provided (Online Resource 5—Supplementary Table 1):

*Enabling timely and flexible access to transplant assessment:* Participants identified the need for a more coordinated and flexible multidisciplinary approach to managing work-up testing and provision of transplant assessment appointments. This would minimise time away from home and expedite waitlisting for potential recipients. Ideas such as “trying to group together assessment investigations and review to prevent need for recurrent travel” and “transplant assessment teams visiting regional areas” were suggested as potential models that would achieve this.

*Aligning communication and education with patient needs:* The need for more appropriate education commencing earlier was highlighted, along with the need for more effective communication between patients (and their families) and clinicians. One participant highlighted “there's no access to education about kidney treatments in the communities” while another sought “improved communication” and “better understanding from nursing staff” while going through the transplant process.

*Reducing financial hardship:* Increased financial support for patients and their families is needed to assist with travel and accommodation expenses, such as “more flexible support from patient assistance transport schemes”. Living expenses while away from home and ongoing costs post-transplant are also an issue. As highlighted by one participant “money is always tight for me, if I could have had the chance to save, but due to cost of living this was pretty much impossible”.

*Fostering comprehensive psychosocial support:* Participants highlighted the complexities around provision of psychological support, as it needs to be provided by both health professionals and culturally appropriate support staff. One participant stated “a nurse navigator type of person for these patients for transplant stuff will in my opinion make a huge impact” with another clarifying “our Indigenous patients need Indigenous Liaison Officer and Indigenous Health Workers to be included”. Some participants highlighted that “having someone to talk to who has already had a transplant would have been helpful”, indicating the sharing of lived experience is of value to other potential recipients.

*Accessing safe and appropriate accommodation:* Transplant recipients identified the need for “accommodation that was suitable to transplant patients” and one felt that “being adequately accommodated is an important part of recovery”.

*Advancing post-transplant provision of care:* The need for faster turnaround of pathology results was highlighted by participants, with one mentioning “currently there is a delay of 1–2 days to have a (tacrolimus) level return”. Streamlining accessibility and cost of medications post-transplant was also highlighted, with one participant suggesting we “nationally standardise the post-op supply of medication”. Participants also expressed a desire for better ongoing medical and psychological support post-transplant, with one wanting “more support with side effects of medication”.

## Discussion

The objective of this study was to determine the experiences of both health professionals and transplant recipients regarding KT processes for regional, rural, and remote populations in Australia, especially in northern Australia. This study provides a unique opportunity to explore the relationship between the experiences, opinions, and attitudes of both health professionals and transplant recipients, whilst identifying priorities with regard to improving access to, and overall experiences of, KT for regional, rural, and remote populations.

The findings of this study indicate that the highest priorities for regional, rural, and remote KT recipients include the information they receive prior to transplant, the financial burden associated with transplant, and medication-related concerns such as potential side effects, number of medications to be taken and access to ongoing medication supply. Patient and health professional participants highlighted the importance of streamlining access to medications locally, the need for increased and earlier education, and reducing the overall financial burden.

Studies have shown that earlier pre-transplant education can improve certain post-transplant outcomes [23], and KDIGO guidelines recommend that all patients expected to reach kidney failure be informed of, educated about, and considered for, transplantation once they reach CKD stage 4 [24]. However delivery of transplant education is complex, and there is an inherent need for flexibility around the way in which education is delivered and to tailor the education to the needs of vulnerable patient populations worldwide [17, 25]. The concerns around when and how transplant education is delivered highlighted in this study are also supported by existing literature. There have been several programs and research studies focused on changing the way education is delivered and developing transplant educational resources and delivery methods that are more appropriate for both Indigenous and other vulnerable patient populations [17, 26].

It was clear in this study that pharmacists believe increased education around transplant medications prior to transplant would be beneficial for patients, and patient participants agreed. However, despite the acknowledgement that receiving medication education in the pre-transplant period is important and beneficial, there seems to be no standardised evidence-based approach to how this should be done, resulting in variability between hospitals and health services [27, 28]. This study also highlights issues around access to medications, indicating that regional, rural, and remote populations may experience difficulties obtaining ongoing supply of transplant medications locally, however this does not appear to be well described in existing literature.

The need for improved psychosocial support was clearly highlighted in this study, particularly the provision of information, mentoring and support to potential transplant recipients from “peer mentors” who have lived experience of KT. The need for health professional navigators and culturally appropriate support staff to be included as part of the transplant team was also identified. The potential benefits of these models of care are also supported by studies across different countries looking at the use of “peer mentors” or “peer navigators” in the KT population from the perspective of both mentors and the mentees, with largely positive outcomes [26, 29]. Some existing services already utilise “peer mentors” as part of the routine KT care provided [30].

The financial burden associated with KT was very clearly identified in this study and has been recognised for many years for the living donor KT cohort specifically [S31]. However, it is only more recently that this has been acknowledged as a significant barrier for regional, rural, and remote patients irrespective of the type of transplant they are receiving [S32]. This supports the findings of this study, with ongoing medication costs and travel and accommodation expenses highlighted as being particularly burdensome. Unfortunately, most of the currently available financial support programs in multiple countries are for living KT donors only [S33, S34].

The need for improved coordination of work-up tests and increased outreach visits by transplant teams to foster timely access to transplant assessment was identified by the health professional participants in this study. There are several studies that have investigated the potential benefits of these services [26, S35]. Offering such models of care would address some of the difficulties with work-up and assessment highlighted in this study, such as reducing time spent away from home, and the need for recurrent travel and the associated financial burden.

Although telehealth is often suggested as a relatively easy, less expensive, and globally relevant model of care to increase access to KT whilst reducing travel burden for patients [S36, S37], the findings from this study indicate the use of telehealth for provision of KT care is still relatively low in Australia. Additionally, this study highlights that the option of telehealth for provision of education and clinical care is not necessarily preferred by patients over face-to-face reviews. This is consistent with other studies looking at KT patient perspectives on the use of telehealth, where reviews have been mixed, and there has been a lack of representation particularly from rural and remote patient populations [S38, S39].

### Limitations

The strengths of this study include the multidisciplinary cross section of health professional participants with different levels of experience and from all categories of remoteness across Australia. This provides a representative sample of KT health professionals to minimise any potential bias and increase transferability of the findings. The inclusion of an open-ended question also added depth and context to the closed question responses, increasing the validity of the findings. However, due to the health service-specific ethics and governance requirements of having consumers as participants in research, the KT recipient participant group was limited to a smaller geographical area. This may therefore limit the transferability of these findings nationally and internationally. The smaller proportion of living donor KT recipient participants may also limit generalisability of the findings to this population. Although a 64% response rate was achieved in the transplant recipient participants, the relatively small sample sizes for both participant groups limited the power of the statistical analysis performed.

## Conclusion

KT recipients from Australian regional, rural, and remote areas face increased barriers and adversities across the entire transplant journey, including pre-transplant testing and assessment and post-transplant care and follow-up. This study indicates that there are multiple aspects of current KT processes in Australia, and particularly northern Australia, that could be optimised to improve patient experiences and clinical outcomes for regional, rural, and remote KT recipients. Future qualitative studies are recommended to explore in depth these findings, and support translation to clinical practice and policy transformation around provision of KT services for this population.

## Supplementary Information

Below is the link to the electronic supplementary material.

Supplementary file1 (PDF 115 KB)

Supplementary file2 (PDF 150 KB)

Supplementary file3 (PDF 1718 KB)

Supplementary file4 (PDF 109 KB)

Supplementary file5 (PDF 157 KB)

Supplementary file6 (PDF 112 KB)

## Data Availability

The datasets generated and analysed during the current study are available from the corresponding author on reasonable request.
