# Supplementary material for: Health professional and transplant recipient perspectives of kidney transplantation in regional, rural, and remote Australia – a survey study
Source: J Nephrol. 2025 Jun 16;38(5):1403–12. doi: 10.1007/s40620-025-02331-4 (PMC12289722; doi:10.1007/s40620-025-02331-4)
Supplement: Supplementary file 1 — Supplementary file1 (PDF 115 KB) [file 40620_2025_2331_MOESM1_ESM.pdf]

Health professional and transplant recipient perspectives of kidney transplantation in regional, rural, and remote Australia – A survey study

Journal of Nephrology

Tara Watters, BPharm (Hons)[1,2], Nicole Scholes-Robertson, PhD[3], Beverley Glass, PhD[1], Andrew J. Mallett, PhD[1,4,5]

[1]*College of Medicine & Dentistry, James Cook University, Townsville, QLD, Australia*
[2]*Department of Renal Medicine, Cairns Hospital, Cairns, QLD, Australia*
[3]*Sydney School of Public Health, The University of Sydney, Sydney NSW, Australia*
[4]*Department of Renal Medicine, Townsville University Hospital, Townsville, QLD, Australia*
[5]*Institute for Molecular Bioscience, The University of Queensland, Brisbane, QLD, Australia*

Correspondence: Tara Watters tara.watters@my.jcu.edu.au

Online Resource 1 – Additional Methods Information

Recruitment

Health Professionals

Participants included a multidisciplinary cross section of health professionals such as nephrologists, transplant nurses, clinical pharmacists, and social workers/Indigenous liaison officers. As the provision of care to kidney transplant recipients in regional, rural, and remote areas of Australia is shared, health professionals based in both metropolitan transplanting centres (providing transplant assessment and peri-transplant care), as well as regional, rural, or remote nephrology or primary care services (providing pre- and post-transplant care) were included. However, to ensure that participants were able to adequately answer the research objective they were only eligible to participate if they had direct involvement in the provision of care to regional, rural, and remote kidney transplant recipients specifically. Health professionals were recruited via invitation to participate disseminated electronically via national and international forums, including Transplantation Society of Australia and New Zealand (TSANZ), Australia and New Zealand Society of Nephrology (ANZSN), Transplant

Nurses' Association (TNA), Renal Society of Australasia (RSA), The Society of Hospital Pharmacists of Australia (SHPA), National Indigenous Kidney Transplant Taskforce (NIKTT), and Australia and New Zealand Dialysis and Transplant Registry (ANZDATA). Participants were also encouraged to partake in snowball recruitment by forwarding the study invitation to eligible health professional colleagues. For sites with Queensland Health ethics and site-specific governance approval (Cairns and Hinterland, Townsville, and Mackay Hospital and Health Services), an email invitation was also disseminated to eligible health professionals within the northern Queensland nephrology departments by a senior staff member in each department. Study invitations were sent out by those professional bodies that agreed to do so at least 3 times over a 3-month period to encourage participation and ensure an adequate response rate was achieved.

Kidney Transplant Recipients

Participants consisted of existing kidney transplant recipients who had received their transplant within the last 5 years residing in regional, rural, or remote areas within northern Queensland. Potential participants were identified through existing public nephrology services within northern Queensland (Cairns and Hinterland, Townsville, and Mackay Hospital and Health Services). An invitation to participate was sent in the form of written participant information via post to 120 eligible patients by a data custodian (usually the transplant coordinator) within these individual hospital and health services. Transplant coordinators were also encouraged to contact potential participants via phone or in person when attending medical appointments to inform them of the study. If no response was received, potential participants were contacted a total of 3 times over a 3-month period, to provide time to consider participation. Posters advertising the study and inviting eligible

patients to participate were also provided to the relevant nephrology services to display in clinic rooms and outpatient waiting areas.

Data Collection

For the health professional participants, 3 different sets of survey questions were designed for specific health professional groups, one for nephrologists, one for clinical pharmacists and one for nursing staff or other, and participants were directed to the relevant set of questions depending on their profession. The surveys were completed by all participants electronically through an online platform (Qualtrics XM Platform, Qualtrics, Seattle United States) or on paper (returned to principal investigator via post) and survey responses were anonymous. Data were collected over a 3-month period from January to March 2024 and remuneration for participants' time was offered in the form of the chance to win a $50 gift voucher for the kidney transplant recipient participants.

Data Analysis

To examine whether demographic attributes and professional background affected agreement with Likert scale survey questions and to compare between responses to different questions, all Likert responses were re-classified into a binary response variable ("agree" vs "disagree"), combined into a single data set for each participant group, and analysed using a mixed effects logistic regression. The random effect was the participant ID, and the fixed effects included the question as well as professional attributes and/or demographic characteristics of each participant. For the health professionals there were fewer "unsure" responses, so these were combined with the "disagree" responses to achieve the binary format. For the kidney transplant recipients, there was a larger proportion (8-25%) of "unsure" responses for most of the questions. In this participant group it was felt that "unsure" was more likely to mean "no

opinion" or indicate that the participant did not understand the question rather than representing a "neutral" response. Therefore all "unsure" responses were excluded (rather than combined with the "disagree" responses) for the logistic regression in the kidney transplant recipient participant group. For all formal tests, a p value <0.05 was used as the criterion for statistical significance.

Qualitative data were imported into NVivo (NVivo, Version 12, Lumivero, Denver United States), and inductive coding used to identify the relevant and recurrent themes. Discussion and refinement of the coding scheme occurred until consensus was reached between the investigators.
