# Supplementary material for: Health professional and transplant recipient perspectives of kidney transplantation in regional, rural, and remote Australia – a survey study
Source: J Nephrol. 2025 Jun 16;38(5):1403–12. doi: 10.1007/s40620-025-02331-4 (PMC12289722; doi:10.1007/s40620-025-02331-4)
Supplement: Supplementary file 2 — Supplementary file2 (PDF 150 KB) [file 40620_2025_2331_MOESM2_ESM.pdf]

Health professional and transplant recipient perspectives of kidney transplantation in regional, rural, and remote Australia – A survey study

Journal of Nephrology

Tara Watters, BPharm (Hons)[1,2], Nicole Scholes-Robertson, PhD[3], Beverley Glass, PhD[1], Andrew J. Mallett, PhD[1,4,5]

[1]College of Medicine & Dentistry, James Cook University, Townsville, QLD, Australia
[2]Department of Renal Medicine, Cairns Hospital, Cairns, QLD, Australia
[3]Sydney School of Public Health, The University of Sydney, Sydney NSW, Australia
[4]Department of Renal Medicine, Townsville University Hospital, Townsville, QLD, Australia
[5]Institute for Molecular Bioscience, The University of Queensland, Brisbane, QLD, Australia

Correspondence: Tara Watters tara.watters@my.jcu.edu.au

Online Resource 2 – Survey Tools

Health Professional Survey Tool

1. In what position are you currently employed in relation to your provision of care to kidney transplant recipients *(select one option)*?

   ☐ Nephrologist at transplanting centre
   ☐ Nephrologist at non-transplanting regional/rural/or remote centre
   ☐ Nursing staff
   ☐ Clinical Pharmacist
   ☐ Social worker or Indigenous liaison officer
   ☐ Other (please specify): ____________________________________

2. How many years have you been working within your profession to provide care to kidney transplant recipients *(select one option)*?

   ☐ 0-7 years
   ☐ 7-14 years
   ☐ >14 years

3. During what part of the kidney transplantation process do you provide care for potential or actual kidney transplant donors or recipients *(select all that apply)*?

   ☐ Pre-transplant (e.g., work-up testing, eligibility assessment)
   ☐ Peri-transplant (including acute post-transplant period)
   ☐ Post-transplant (excluding acute post-transplant period)
   ☐ All of the above

4. Which of the following service delivery models do you currently utilise for provision of care to potential or actual kidney transplant donors or recipients *(select all that apply)*?

   ☐ Face-to-face appointments
   ☐ Telephone appointments
   ☐ Telehealth (i.e., videoconference) appointments for **pre-transplant care**
   ☐ Telehealth (i.e., videoconference) appointments for **post-transplant care**
   ☐ Other (please specify): ____________________________________

5. What is the location of your primary place of practice?

   Please provide name of city/town AND the post code: ____________________________

QUESTIONS 6 ONWARDS DEPENDS ON WHICH OPTION IS SELECTED IN Q1

<u>Nephrologists</u>

6. Do you discuss the option of living kidney donor transplant with all potential kidney transplant recipients *(select one option)*?

   ☐ Yes
   ☐ No

7. **Please rate the following statements using the scale provided according to your own professional experience *(circle one option)*:**

A. Targeted education to increase awareness around both deceased and living donor kidney transplantation as a treatment option would increase transplantation rates in regional/rural/remote patient populations.

Strongly Agree Agree Unsure Disagree Strongly Disagree

B. Provision of pre-transplant care (e.g., eligibility assessment) and education via a telehealth service modality would increase access to transplant for patients in regional/rural/remote areas.

Strongly Agree Agree Unsure Disagree Strongly Disagree

C. I take into consideration compliance with dialysis and medications when deciding whether to refer and/or assessing a patients' eligibility to receive a kidney transplant.

Strongly Agree Agree Unsure Disagree Strongly Disagree

D. Receiving psychosocial support or mentoring from peers (e.g., patient navigators, support groups) would be beneficial for potential transplant recipients from regional/rural/remote areas.

Strongly Agree *Agree* *Unsure* *Disagree* *Strongly Disagree*

E. There is a balance between the potential benefits of a kidney transplant for an individual patient versus maximising the utility of donor organs when determining a patients' eligibility to receive a transplant.

Strongly Agree *Agree* *Unsure* *Disagree* *Strongly Disagree*

F. The development of an agreed national approach to patient selection, eligibility criteria and organ distribution would address inequities in access to transplant for regional/rural/remote patient populations.

Strongly Agree *Agree* *Unsure* *Disagree* *Strongly Disagree*

G. The use of point-of-care testing for therapeutic drug monitoring of immunosuppressants would be beneficial for kidney transplant patients in regional/rural/remote areas.

Strongly Agree *Agree* *Unsure* *Disagree* *Strongly Disagree*

<u>Clinical Pharmacists</u>

6. Please rate the following statements using the scale provided according to your own professional experience *(circle one option)*:

A. Increased provision of information about transplant medications (e.g., number of tablets, possible side effects) as part of the transplant work-up process would be beneficial for patients.

Strongly Agree *Agree* *Unsure* *Disagree* *Strongly Disagree*

B. Financial support (e.g., subsidising or waiving patient co-payments) would be beneficial for kidney transplant recipients from regional/rural/remote areas.

Strongly Agree *Agree* *Unsure* *Disagree* *Strongly Disagree*

C. Regional/rural/remote kidney transplant recipients often experience difficulties related to obtaining ongoing supply of medications post-transplant.

Strongly Agree *Agree* *Unsure* *Disagree* *Strongly Disagree*

D. The use of point-of-care testing for therapeutic drug monitoring of immunosuppressants would be beneficial for kidney transplant patients in regional/rural/remote areas.

Strongly Agree *Agree* *Unsure* *Disagree* *Strongly Disagree*

<u>Nursing staff & other health professionals</u>

6. Please rate the following statements using the scale provided according to your own professional experience *(circle one option)*:

A. Targeted education to increase awareness around both deceased and living donor kidney transplantation as a treatment option would increase transplantation rates in regional/rural/remote patient populations.

Strongly Agree *Agree* *Unsure* *Disagree* *Strongly Disagree*

B. Provision of pre-transplant care (e.g., eligibility assessment) and education via a telehealth service modality would increase access to transplant for patients in regional/rural/remote areas.

Strongly Agree *Agree* *Unsure* *Disagree* *Strongly Disagree*

C. Receiving psychosocial support or mentoring from peers (e.g., patient navigators, support groups) would be beneficial for potential transplant recipients from regional/rural/remote areas.

Strongly Agree *Agree* *Unsure* *Disagree* *Strongly Disagree*

D. Financial support (e.g., subsidising or waiving patient travel and accommodation costs) would be beneficial for kidney transplant recipients from regional/rural/remote areas undergoing transplant work-up or post-transplant.

Strongly Agree *Agree* *Unsure* *Disagree* *Strongly Disagree*

7/8 (All Participants). If there was one thing you could change about current kidney transplantation processes that would improve the experiences or clinical outcomes for patients from regional/rural/remote areas, what would it be (provide response below)?

____

____

____

Kidney Transplant Recipient Survey

For questions 1 – 3 please select one option only by marking the relevant box with a tick or cross.

1. What type of kidney transplant did you receive?

☐ Deceased donor kidney
☐ Living related kidney donor (i.e. from a living relative)
☐ Living unrelated kidney donor (e.g. from a partner, friend)
☐ Unsure

2. How long had you been on dialysis for prior to receiving your kidney transplant?

☐ I was not having dialysis treatment when I received my kidney transplant
☐ 0 – 1 year
☐ 1 – 3 years
☐ 3 – 5 years
☐ >5 years

3. Do you identify as Aboriginal and/or Torres Strait Islander?

☐ Aboriginal
☐ Torres Strait Islander
☐ Both
☐ Neither
☐ Prefer not to say

4. Prior to starting on dialysis, where were you living?

Please provide name of town AND the post code (if known): _______________________

For questions 5 - 7 please select one option only by marking the relevant box with a tick or cross.

5. The option of receiving a kidney donated from a living relative or other living person was discussed with me.

☐ Yes
☐ No
☐ Unsure

6. I received information about what would be involved for me receiving a kidney transplantation (e.g., travel requirements, approximate out-of-pocket costs, medical tests required) prior to receiving my kidney transplant.

   ☐ Yes
   ☐ No
   ☐ Unsure

7. I received information about the medications I would be taking after the transplant (e.g., number of tablets, possible side effects), prior to receiving my kidney transplant.

   ☐ Yes
   ☐ No
   ☐ Unsure

8. Please rate the following statements using the scale provided according to your own kidney transplant experience *(circle one option)*:

A. The time I had to wait to receive a kidney transplant was reasonable.

Strongly Agree   *Agree*   *Unsure*   *Disagree*   *Strongly Disagree*

B. The fact that I live in a regional / rural / remote area away from the transplant hospital in Brisbane increased my waiting time for a kidney transplant.

Strongly Agree   *Agree*   *Unsure*   *Disagree*   *Strongly Disagree*

C. Communicating with the kidney / transplant health professionals (e.g., doctors, nurses, pharmacists, or other staff) was easy.

Strongly Agree   *Agree*   *Unsure*   *Disagree*   *Strongly Disagree*

D. The time associated with travelling to attend appointments and have tests done <u>prior to</u> receiving my kidney transplant had a negative effect on me and/or my family.

Strongly Agree   *Agree*   *Unsure*   *Disagree*   *Strongly Disagree*

E. The financial costs associated with travelling to attend appointments and have tests done <u>prior to</u> receiving my kidney transplant had a negative effect on me and/or my family.

Strongly Agree   *Agree*   *Unsure*   *Disagree*   *Strongly Disagree*

F. Being separated from my family, friends, or support network to attend appointments and have tests done prior to receiving my kidney transplant **did not** have any effect on me and/or my family.

Strongly Agree *Agree* *Unsure* *Disagree* *Strongly Disagree*

G. I would have preferred to receive information and education about kidney transplantation via videoconference rather than travelling.

Strongly Agree *Agree* *Unsure* *Disagree* *Strongly Disagree*

H. I would have preferred to attend medical appointments prior to receiving my kidney transplant via videoconference rather than travelling for in-person appointments.

Strongly Agree *Agree* *Unsure* *Disagree* *Strongly Disagree*

I. Receiving information, support or mentoring from other patients who have already received a kidney transplant would have made my own transplant experience easier / better.

Strongly Agree *Agree* *Unsure* *Disagree* *Strongly Disagree*

J. The financial cost associated with ongoing medication supply following my kidney transplant had a negative effect on me and/or my family.

Strongly Agree *Agree* *Unsure* *Disagree* *Strongly Disagree*

K. I have experienced difficulties related to obtaining ongoing supply of my medications following my kidney transplant.

Strongly Agree *Agree* *Unsure* *Disagree* *Strongly Disagree*

9. Regarding your kidney transplant experience, please rank the following in order of importance to you by numbering options provided from 1 – 10 (with 1 being the MOST important and 10 being the LEAST important). Please only use each number ONCE:

___ Access to transport to attend appointments

___ Accommodation when attending appointments

___ High financial costs incurred related to treatment

___ Separation from family / social support

___ Leave / absence from employment

___ Assistance or support with communicating with health professionals

___ Information prior to transplant

___ Large number of medications to be taken

___ Potential medication side effects

___ Access to ongoing medication supply

10. If there was one thing you could change about your own personal kidney transplant experience, what would it be (provide response below)?

____

____

____

____

____

____

____
