# Supplementary material for: Health professional and transplant recipient perspectives of kidney transplantation in regional, rural, and remote Australia – a survey study
Source: J Nephrol. 2025 Jun 16;38(5):1403–12. doi: 10.1007/s40620-025-02331-4 (PMC12289722; doi:10.1007/s40620-025-02331-4)
Supplement: Supplementary file 3 — Supplementary file3 (PDF 1718 KB) [file 40620_2025_2331_MOESM3_ESM.pdf]

Health professional and transplant recipient perspectives of kidney transplantation in regional, rural, and remote Australia – A survey study

Journal of Nephrology

Tara Watters, BPharm (Hons)[1,2], Nicole Scholes-Robertson, PhD[3], Beverley Glass, PhD[1], Andrew J. Mallett, PhD[1,4,5]

[1]*College of Medicine & Dentistry, James Cook University, Townsville, QLD, Australia*
[2]*Department of Renal Medicine, Cairns Hospital, Cairns, QLD, Australia*
[3]*Sydney School of Public Health, The University of Sydney, Sydney NSW, Australia*
[4]*Department of Renal Medicine, Townsville University Hospital, Townsville, QLD, Australia*
[5]*Institute for Molecular Bioscience, The University of Queensland, Brisbane, QLD, Australia*

Correspondence: Tara Watters tara.watters@my.jcu.edu.au

Online Resource 3 – SPSS Output

Health professional participants – tests of association analysis

Crosstab

			Abb - Targeted education to increase awareness around both deceased and living donor kidney transplantation as a treatment option would increase transplantation rates in regional/rural/remote patient populations			
			Disagree / Strongly Disagree	Unsure	Agree / Strongly Agree	Total
Profession	Nephrologist at transplanting centre	Count	0	0	15	15
		Expected Count	1.5	1.0	12.5	15.0
		% within Profession	0.0%	0.0%	100.0%	100.0%
		% within Abb - Targeted education to increase awareness around both deceased and living donor kidney transplantation as a treatment option would increase transplantation rates in regional/rural/remote patient populations	0.0%	0.0%	60.0%	50.0%
		% of Total	0.0%	0.0%	50.0%	50.0%
	Nephrologist at non-transplanting regional/rural/or remote centre	Count	3	2	10	15
		Expected Count	1.5	1.0	12.5	15.0
		% within Profession	20.0%	13.3%	66.7%	100.0%
		% within Abb - Targeted education to increase awareness around both deceased and living donor kidney transplantation as a treatment option would increase transplantation rates in regional/rural/remote patient populations	100.0%	100.0%	40.0%	50.0%
		% of Total	10.0%	6.7%	33.3%	50.0%
Total		Count	3	2	25	30
		Expected Count	3.0	2.0	25.0	30.0
		% within Profession	10.0%	6.7%	83.3%	100.0%
		% within Abb - Targeted education to increase awareness around both deceased and living	100.0%	100.0%	100.0%	100.0%

Chi-Square Tests

	Value	df	Asymptotic Significance (2-sided)	Exact Sig. (2-sided)	Exact Sig. (1-sided)	Point Probability
Pearson Chi-Square	6.000[a]	2	.050	.042		
Likelihood Ratio	7.938	2	.019	.042		
Fisher-Freeman-Halton Exact Test	5.207			.042		
Linear-by-Linear Association	5.213[b]	1	.022	.042	.021	.021
N of Valid Cases	30					

a. 4 cells (66.7%) have expected count less than 5. The minimum expected count is 1.00.

b. The standardized statistic is -2.283.

Crosstab

			Abb - There is a balance between the potential benefits of a kidney transplant for an individual patient versus maximising the utility of donor organs when determining a patients' eligibility to receive a transplant			
			Disagree / Strongly Disagree	Unsure	Agree / Strongly Agree	Total
Abb - Years of Experience	< 7 years	Count	3	1	7	11
		Expected Count	1.1	.4	9.5	11.0
		% within Abb - Years of Experience	27.3%	9.1%	63.6%	100.0%
		% within Abb - There is a balance between the potential benefits of a kidney transplant for an individual patient versus maximising the utility of donor organs when determining a patients' eligibility to receive a transplant	100.0%	100.0%	26.9%	36.7%
		% of Total	10.0%	3.3%	23.3%	36.7%
	> 7 years	Count	0	0	19	19
		Expected Count	1.9	.6	16.5	19.0
		% within Abb - Years of Experience	0.0%	0.0%	100.0%	100.0%
		% within Abb - There is a balance between the potential benefits of a kidney transplant for an individual patient versus maximising the utility of donor organs when determining a patients' eligibility to receive a transplant	0.0%	0.0%	73.1%	63.3%
		% of Total	0.0%	0.0%	63.3%	63.3%
Total		Count	3	1	26	30
		Expected Count	3.0	1.0	26.0	30.0
		% within Abb - Years of Experience	10.0%	3.3%	86.7%	100.0%
		% within Abb - There is a balance between the [illegible]	100.0%	100.0%	100.0%	100.0%

Chi-Square Tests

	Value	df	Asymptotic Significance (2-sided)	Exact Sig. (2-sided)	Exact Sig. (1-sided)	Point Probability
Pearson Chi-Square	7.972[a]	2	.019	.012		
Likelihood Ratio	9.140	2	.010	.012		
Fisher-Freeman-Halton Exact Test	7.128			.012		
Linear-by-Linear Association	7.198[b]	1	.007	.012	.012	.012
N of Valid Cases	30					

a. 4 cells (66.7%) have expected count less than 5. The minimum expected count is .37.

b. The standardized statistic is 2.683.

Crosstab

			Abb - Regional/rural/remote kidney transplant recipients often experience difficulties related to obtaining ongoing supply of medications post-transplant			
			Disagree / Strongly Disagree	Unsure	Agree / Strongly Agree	Total
Abb2 - Rurality of principle place of practice	Metropolitan Area	Count	1	3	2	6
		Expected Count	.4	1.2	4.4	6.0
		% within Abb2 - Rurality of principle place of practice	16.7%	50.0%	33.3%	100.0%
		% within Abb - Regional/rural/remote kidney transplant recipients often experience difficulties related to obtaining ongoing supply of medications post-transplant	100.0%	100.0%	18.2%	40.0%
		% of Total	6.7%	20.0%	13.3%	40.0%
	Regional / Rural / Remote Area	Count	0	0	9	9
		Expected Count	.6	1.8	6.6	9.0
		% within Abb2 - Rurality of principle place of practice	0.0%	0.0%	100.0%	100.0%
		% within Abb - Regional/rural/remote kidney transplant recipients often experience difficulties related to obtaining ongoing supply of medications post-transplant	0.0%	0.0%	81.8%	60.0%
		% of Total	0.0%	0.0%	60.0%	60.0%
Total		Count	1	3	11	15
		Expected Count	1.0	3.0	11.0	15.0
		% within Abb2 - Rurality of principle place of practice	6.7%	20.0%	73.3%	100.0%

Chi-Square Tests

	Value	df	Asymptotic Significance (2-sided)	Exact Sig. (2-sided)	Exact Sig. (1-sided)	Point Probability
Pearson Chi-Square	8.182[a]	2	.017	.011		
Likelihood Ratio	9.759	2	.008	.011		
Fisher-Freeman-Halton Exact Test	7.412			.011		
Linear-by-Linear Association	6.563[b]	1	.010	.011	.011	.011
N of Valid Cases	15					

a. 5 cells (83.3%) have expected count less than 5. The minimum expected count is .40.

b. The standardized statistic is 2.562.

Health professional participants (nephrologists + nurses / other health professionals) - mixed logistic regression analysis

Fixed Effects[a]

Source	F	df1	df2	Sig.
Corrected Model	3.873	7	20	.008
Prof	4.152	3	20	.019
AbbExperience	9.588	1	20	.006
Question	3.840	3	20	.025

Probability distribution: Binomial
Link function: Logit [a]

a. Target: Binary Response

Fixed Coefficients[a]

Model Term	Coefficient	Std. Error	t	Sig.	95% Confidence Interval		Exp (Coefficient)	95% Confidence Interval for Exp(Coefficient)	
					Lower	Upper		Lower	Upper
Intercept	-1.948	.8215	-2.371	.028	-3.661	-.234	.143	.026	.791
Prof=1	-1.170	.6192	-1.889	.073	-2.461	.122	.310	.085	1.130
Prof=2	.176	.5134	.343	.735	-.895	1.247	1.192	.409	3.479
Prof=4	-1.653	.6610	-2.500	.021	-3.031	-.274	.192	.048	.760
Prof=5	0[b]	.	.	.	.	.	.	.	.
AbbExperience=1	-1.498	.4839	-3.096	.006	-2.508	-.489	.223	.081	.613
AbbExperience=2	0[b]	.	.	.	.	.	.	.	.
Question=7A	1.135	.7529	1.507	.147	-.436	2.705	3.111	.647	14.961
Question=7B	1.521	.7420	2.049	.054	-.027	3.068	4.575	.973	21.504
Question=7D	-.588	.9227	-.637	.531	-2.513	1.337	.556	.081	3.807
Question=7G	0[b]	.	.	.	.	.	.	.	.

Probability distribution: Binomial
Link function: Logit [a]

a. Target: Binary Response

b. This coefficient is set to zero because it is redundant.

Kidney transplant recipients – tests of association analysis

			The option of receiving a kidney donated from a living relative or other living person was discussed with me		
			Yes	No	Total
Abb - Aboriginal and/or Torres Strait Islander	Aboriginal and/or Torres Strait Islander	Count	9	6	15
		Expected Count	12.4	2.6	15.0
		% within Abb - Aboriginal and/or Torres Strait Islander	60.0%	40.0%	100.0%
		% within The option of receiving a kidney donated from a living relative or other living person was discussed with me	17.0%	54.5%	23.4%
		% of Total	14.1%	9.4%	23.4%
	Non Indigenous	Count	44	5	49
		Expected Count	40.6	8.4	49.0
		% within Abb - Aboriginal and/or Torres Strait Islander	89.8%	10.2%	100.0%
		% within The option of receiving a kidney donated from a living relative or other living person was discussed with me	83.0%	45.5%	76.6%
		% of Total	68.8%	7.8%	76.6%
Total		Count	53	11	64
		Expected Count	53.0	11.0	64.0
		% within Abb - Aboriginal and/or Torres Strait Islander	82.8%	17.2%	100.0%
		% within The option of receiving a kidney donated from a living relative or other living person was discussed with me	100.0%	100.0%	100.0%
		% of Total	82.8%	17.2%	100.0%

Chi-Square Tests

	Value	df	Asymptotic Significance (2-sided)	Exact Sig. (2-sided)	Exact Sig. (1-sided)	Point Probability
Pearson Chi-Square	7.163[a]	1	.007	.015	.015	
Continuity Correction[b]	5.223	1	.022			
Likelihood Ratio	6.247	1	.012	.054	.015	
Fisher's Exact Test				.015	.015	
Linear-by-Linear Association	7.051[c]	1	.008	.015	.015	.013
N of Valid Cases	64					

a. 1 cells (25.0%) have expected count less than 5. The minimum expected count is 2.58.

b. Computed only for a 2x2 table

c. The standardized statistic is -2.655.

			Abb - The time I had to wait to receive a kidney transplant was reasonable			
			Strongly Disagree / Disagree	Unsure	Agree / Strongly Agree	Total
Abb - Duration of dialysis treatment at time of transplant	0 - 3 years	Count	2	2	46	50
		Expected Count	6.5	3.2	40.3	50.0
		% within Abb - Duration of dialysis treatment at time of transplant	4.0%	4.0%	92.0%	100.0%
		% within Abb - The time I had to wait to receive a kidney transplant was reasonable	20.0%	40.0%	74.2%	64.9%
		% of Total	2.6%	2.6%	59.7%	64.9%
	> 3 years	Count	8	3	16	27
		Expected Count	3.5	1.8	21.7	27.0
		% within Abb - Duration of dialysis treatment at time of transplant	29.6%	11.1%	59.3%	100.0%
		% within Abb - The time I had to wait to receive a kidney transplant was reasonable	80.0%	60.0%	25.8%	35.1%
		% of Total	10.4%	3.9%	20.8%	35.1%
Total		Count	10	5	62	77
		Expected Count	10.0	5.0	62.0	77.0
		% within Abb - Duration of dialysis treatment at time of transplant	13.0%	6.5%	80.5%	100.0%
		% within Abb - The time I had to wait to receive a kidney transplant was reasonable	100.0%	100.0%	100.0%	100.0%
		% of Total	13.0%	6.5%	80.5%	100.0%

Chi-Square Tests

	Value	df	Asymptotic Significance (2-sided)	Exact Sig. (2-sided)	Exact Sig. (1-sided)	Point Probability
Pearson Chi-Square	12.567[a]	2	.002	<.001		
Likelihood Ratio	12.224	2	.002	.003		
Fisher-Freeman-Halton Exact Test	11.946			<.001		
Linear-by-Linear Association	12.309[b]	1	<.001	<.001	<.001	.000
N of Valid Cases	77					

a. 3 cells (50.0%) have expected count less than 5. The minimum expected count is 1.75.

b. The standardized statistic is -3.508.

Abb - Aboriginal and/or Torres Strait Islander * Abb - Duration of dialysis treatment at time of transplant Crosstabulation

			Abb - Duration of dialysis treatment at time of transplant		
			0 - 3 years	> 3 years	Total
Abb - Aboriginal and/or Torres Strait Islander	Aboriginal and/or Torres Strait Islander	Count	9	10	19
		Expected Count	12.5	6.5	19.0
		% within Abb - Aboriginal and/or Torres Strait Islander	47.4%	52.6%	100.0%
		% within Abb - Duration of dialysis treatment at time of transplant	18.8%	40.0%	26.0%
		% of Total	12.3%	13.7%	26.0%
	Non Indigenous	Count	39	15	54
		Expected Count	35.5	18.5	54.0
		% within Abb - Aboriginal and/or Torres Strait Islander	72.2%	27.8%	100.0%
		% within Abb - Duration of dialysis treatment at time of transplant	81.3%	60.0%	74.0%
		% of Total	53.4%	20.5%	74.0%
Total		Count	48	25	73
		Expected Count	48.0	25.0	73.0
		% within Abb - Aboriginal and/or Torres Strait Islander	65.8%	34.2%	100.0%
		% within Abb - Duration of dialysis treatment at time of transplant	100.0%	100.0%	100.0%
		% of Total	65.8%	34.2%	100.0%

Chi-Square Tests

	Value	df	Asymptotic Significance (2-sided)	Exact Sig. (2-sided)	Exact Sig. (1-sided)
Pearson Chi-Square	3.855[a]	1	.050		
Continuity Correction[b]	2.831	1	.092		
Likelihood Ratio	3.730	1	.053		
Fisher's Exact Test				.090	.048
Linear-by-Linear Association	3.803	1	.051		
N of Valid Cases	73				

a. 0 cells (0.0%) have expected count less than 5. The minimum expected count is 6.51.

b. Computed only for a 2x2 table

			Abb - The fact that I live in a regional / rural / remote area away from the transplant hospital in Brisbane increased my waiting time for a kidney transplant			
			Strongly Disagree / Disagree	Unsure	Agree / Strongly Agree	Total
Abb - Aboriginal and/or Torres Strait Islander	Aboriginal and/or Torres Strait Islander	Count	5	5	9	19
		Expected Count	9.9	4.7	4.4	19.0
		% within Abb - Aboriginal and/or Torres Strait Islander	26.3%	26.3%	47.4%	100.0%
		% within Abb - The fact that I live in a regional / rural / remote area away from the transplant hospital in Brisbane increased my waiting time for a kidney transplant	13.2%	27.8%	52.9%	26.0%
		% of Total	6.8%	6.8%	12.3%	26.0%
	Non Indigenous	Count	33	13	8	54
		Expected Count	28.1	13.3	12.6	54.0
		% within Abb - Aboriginal and/or Torres Strait Islander	61.1%	24.1%	14.8%	100.0%
		% within Abb - The fact that I live in a regional / rural / remote area away from the transplant hospital in Brisbane increased my waiting time for a kidney transplant	86.8%	72.2%	47.1%	74.0%
		% of Total	45.2%	17.8%	11.0%	74.0%
Total		Count	38	18	17	73
		Expected Count	38.0	18.0	17.0	73.0
		% within Abb - Aboriginal and/or Torres Strait Islander	52.1%	24.7%	23.3%	100.0%
		% within Abb - The fact that I live in a regional / rural / remote area away from the transplant hospital in Brisbane increased my waiting time for a kidney transplant	100.0%	100.0%	100.0%	100.0%
		% of Total	52.1%	24.7%	23.3%	100.0%

Chi-Square Tests

	Value	df	Asymptotic Significance (2-sided)	Exact Sig. (2-sided)	Exact Sig. (1-sided)	Point Probability
Pearson Chi-Square	9.693[a]	2	.008	.007		
Likelihood Ratio	9.337	2	.009	.012		
Fisher-Freeman-Halton Exact Test	9.205			.011		
Linear-by-Linear Association	9.375[b]	1	.002	.003	.002	.001
N of Valid Cases	73					

a. 2 cells (33.3%) have expected count less than 5. The minimum expected count is 4.42.

b. The standardized statistic is -3.062.

Abb - Aboriginal and/or Torres Strait Islander * Abb2 - Degree of Rurality Crosstabulation

			Abb2 - Degree of Rurality		
			Metropolitan & Regional Areas	Rural & Remote Areas	Total
Abb - Aboriginal and/or Torres Strait Islander	Aboriginal and/or Torres Strait Islander	Count	7	12	19
		Expected Count	13.3	5.7	19.0
		% within Abb - Aboriginal and/or Torres Strait Islander	36.8%	63.2%	100.0%
		% within Abb2 - Degree of Rurality	13.7%	54.5%	26.0%
		% of Total	9.6%	16.4%	26.0%
	Non Indigenous	Count	44	10	54
		Expected Count	37.7	16.3	54.0
		% within Abb - Aboriginal and/or Torres Strait Islander	81.5%	18.5%	100.0%
		% within Abb2 - Degree of Rurality	86.3%	45.5%	74.0%
		% of Total	60.3%	13.7%	74.0%
Total		Count	51	22	73
		Expected Count	51.0	22.0	73.0
		% within Abb - Aboriginal and/or Torres Strait Islander	69.9%	30.1%	100.0%
		% within Abb2 - Degree of Rurality	100.0%	100.0%	100.0%
		% of Total	69.9%	30.1%	100.0%

Chi-Square Tests

	Value	df	Asymptotic Significance (2-sided)	Exact Sig. (2-sided)	Exact Sig. (1-sided)
Pearson Chi-Square	13.302[a]	1	<.001		
Continuity Correction[b]	11.266	1	<.001		
Likelihood Ratio	12.597	1	<.001		
Fisher's Exact Test				<.001	<.001
Linear-by-Linear Association	13.120	1	<.001		
N of Valid Cases	73				

a. 0 cells (0.0%) have expected count less than 5. The minimum expected count is 5.73.

b. Computed only for a 2x2 table

			Abb - The time associated with travelling to attend appointments and have tests done prior to receiving my kidney transplant had a negative effect on me and/or my family			
			Strongly Disagree / Disagree	Unsure	Agree / Strongly Agree	Total
Abb - Aboriginal and/or Torres Strait Islander	Aboriginal and/or Torres Strait Islander	Count	9	5	5	19
		Expected Count	12.5	1.8	4.7	19.0
		% within Abb - Aboriginal and/or Torres Strait Islander	47.4%	26.3%	26.3%	100.0%
		% within Abb - The time associated with travelling to attend appointments and have tests done prior to receiving my kidney transplant had a negative effect on me and/or my family	18.8%	71.4%	27.8%	26.0%
		% of Total	12.3%	6.8%	6.8%	26.0%
	Non Indigenous	Count	39	2	13	54
		Expected Count	35.5	5.2	13.3	54.0
		% within Abb - Aboriginal and/or Torres Strait Islander	72.2%	3.7%	24.1%	100.0%
		% within Abb - The time associated with travelling to attend appointments and have tests done prior to receiving my kidney transplant had a negative effect on me and/or my family	81.3%	28.6%	72.2%	74.0%
		% of Total	53.4%	2.7%	17.8%	74.0%
Total		Count	48	7	18	73
		Expected Count	48.0	7.0	18.0	73.0
		% within Abb - Aboriginal and/or Torres Strait Islander	65.8%	9.6%	24.7%	100.0%
		% within Abb - The time associated with travelling to attend appointments and have tests done prior to receiving my kidney transplant had a negative effect on me and/or my family	100.0%	100.0%	100.0%	100.0%
		% of Total	65.8%	9.6%	24.7%	100.0%

Chi-Square Tests

	Value	df	Asymptotic Significance (2-sided)	Exact Sig. (2-sided)	Exact Sig. (1-sided)	Point Probability
Pearson Chi-Square	8.843[a]	2	.012	.011		
Likelihood Ratio	7.735	2	.021	.024		
Fisher-Freeman-Halton Exact Test	7.739			.012		
Linear-by-Linear Association	1.384[b]	1	.239	.281	.154	.060
N of Valid Cases	73					

a. 2 cells (33.3%) have expected count less than 5. The minimum expected count is 1.82.

b. The standardized statistic is -1.177.

			Abb - The time associated with travelling to attend appointments and have tests done prior to receiving my kidney transplant had a negative effect on me and/or my family			
			Strongly Disagree / Disagree	Unsure	Agree / Strongly Agree	Total
Abb2 - Degree of Rurality	Metropolitan & Regional Areas	Count	38	2	14	54
		Expected Count	34.4	5.6	14.0	54.0
		% within Abb2 - Degree of Rurality	70.4%	3.7%	25.9%	100.0%
		% within Abb - The time associated with travelling to attend appointments and have tests done prior to receiving my kidney transplant had a negative effect on me and/or my family	77.6%	25.0%	70.0%	70.1%
		% of Total	49.4%	2.6%	18.2%	70.1%
	Rural & Remote Areas	Count	11	6	6	23
		Expected Count	14.6	2.4	6.0	23.0
		% within Abb2 - Degree of Rurality	47.8%	26.1%	26.1%	100.0%
		% within Abb - The time associated with travelling to attend appointments and have tests done prior to receiving my kidney transplant had a negative effect on me and/or my family	22.4%	75.0%	30.0%	29.9%
		% of Total	14.3%	7.8%	7.8%	29.9%
Total		Count	49	8	20	77
		Expected Count	49.0	8.0	20.0	77.0
		% within Abb2 - Degree of Rurality	63.6%	10.4%	26.0%	100.0%
		% within Abb - The time associated with travelling to attend appointments and have tests done prior to receiving my kidney transplant had a negative effect on me and/or my family	100.0%	100.0%	100.0%	100.0%
		% of Total	63.6%	10.4%	26.0%	100.0%

Chi-Square Tests

	Value	df	Asymptotic Significance (2-sided)	Exact Sig. (2-sided)	Exact Sig. (1-sided)	Point Probability
Pearson Chi-Square	9.067[a]	2	.011	.009		
Likelihood Ratio	8.283	2	.016	.023		
Fisher-Freeman-Halton Exact Test	8.118			.013		
Linear-by-Linear Association	1.088[b]	1	.297	.321	.184	.065
N of Valid Cases	77					

a. 1 cells (16.7%) have expected count less than 5. The minimum expected count is 2.39.

b. The standardized statistic is 1.043.

Crosstab

			Abb - The financial costs associated with travelling to attend appointments and have tests done prior to receiving my kidney transplant had a negative effect on me and/or my family			
			Strongly Disagree / Disagree	Unsure	Agree / Strongly Agree	Total
Abb - Type of Transplant	Deceased Donor	Count	37	7	19	63
		Expected Count	34.1	7.0	21.9	63.0
		% within Abb - Type of Transplant	58.7%	11.1%	30.2%	100.0%
		% within Abb - The financial costs associated with travelling to attend appointments and have tests done prior to receiving my kidney transplant had a negative effect on me and/or my family	94.9%	87.5%	76.0%	87.5%
		% of Total	51.4%	9.7%	26.4%	87.5%
	Living Donor	Count	2	1	6	9
		Expected Count	4.9	1.0	3.1	9.0
		% within Abb - Type of Transplant	22.2%	11.1%	66.7%	100.0%
		% within Abb - The financial costs associated with travelling to attend appointments and have tests done prior to receiving my kidney transplant had a negative effect on me and/or my family	5.1%	12.5%	24.0%	12.5%
		% of Total	2.8%	1.4%	8.3%	12.5%
Total		Count	39	8	25	72
		Expected Count	39.0	8.0	25.0	72.0
		% within Abb - Type of Transplant	54.2%	11.1%	34.7%	100.0%
		% within Abb - The financial costs associated with travelling to attend appointments and have tests done prior to receiving my kidney transplant had a negative effect on me and/or my family	100.0%	100.0%	100.0%	100.0%
		% of Total	54.2%	11.1%	34.7%	100.0%

Chi-Square Tests

	Value	df	Asymptotic Significance (2-sided)	Exact Sig. (2-sided)	Exact Sig. (1-sided)	Point Probability
Pearson Chi-Square	4.961[a]	2	.084	.128		
Likelihood Ratio	4.895	2	.086	.098		
Fisher-Freeman-Halton Exact Test	4.856			.061		
Linear-by-Linear Association	4.865[b]	1	.027	.031	.023	.014
N of Valid Cases	72					

a. 3 cells (50.0%) have expected count less than 5. The minimum expected count is 1.00.

b. The standardized statistic is 2.206.

Kidney transplant recipients – mixed logistic regression analysis

Fixed Effects[a]

Source	F	df1	df2	Sig.
Corrected Model	20.543	11	10	<.001
MMMAbb2	8.148	1	10	.017
Question	22.156	10	10	<.001

Probability distribution: Binomial
Link function: Logit [a]

a. Target: Binary Response

Fixed Coefficients[a]

Model Term	Coefficient	Std. Error	t	Sig.	95% Confidence Interval		Exp (Coefficient)	95% Confidence Interval for Exp(Coefficient)	
					Lower	Upper		Lower	Upper
Intercept	-.670	.2165	-3.094	.011	-1.152	-.188	.512	.316	.829
MMMAbb2=1	-.398	.1396	-2.854	.017	-.709	-.087	.671	.492	.916
MMMAbb2=2	0[b]	.	.	.	.	.	.	.	.
Question=8A	-.884	.3237	-2.732	.021	-1.606	-.163	.413	.201	.849
Question=8B	.079	.2933	.269	.793	-.575	.732	1.082	.563	2.080
Question=8C	-1.536	.3765	-4.079	.002	-2.375	-.697	.215	.093	.498
Question=8D	.068	.2805	.243	.813	-.557	.693	1.071	.573	2.000
Question=8E	.524	.2711	1.934	.082	-.080	1.129	1.690	.923	3.091
Question=8F	.053	.2772	.190	.853	-.565	.670	1.054	.568	1.954
Question=8G	1.953	.2861	6.826	<.001	1.315	2.590	7.050	3.726	13.336
Question=8H	2.143	.2914	7.353	<.001	1.493	2.792	8.523	4.452	16.315
Question=8I	-.771	.3256	-2.367	.039	-1.496	-.045	.463	.224	.956
Question=8J	.923	.2657	3.472	.006	.331	1.515	2.516	1.392	4.548
Question=8K	0[b]	.	.	.	.	.	.	.	.

Probability distribution: Binomial
Link function: Logit [a]

a. Target: Binary Response

b. This coefficient is set to zero because it is redundant.
