# Supplementary material for: Health professional and transplant recipient perspectives of kidney transplantation in regional, rural, and remote Australia – a survey study
Source: J Nephrol. 2025 Jun 16;38(5):1403–12. doi: 10.1007/s40620-025-02331-4 (PMC12289722; doi:10.1007/s40620-025-02331-4)
Supplement: Supplementary file 4 — Supplementary file4 (PDF 109 KB) [file 40620_2025_2331_MOESM4_ESM.pdf]

Health professional and transplant recipient perspectives of kidney transplantation in regional, rural, and remote Australia – A survey study

Journal of Nephrology

Tara Watters, BPharm (Hons)[1,2], Nicole Scholes-Robertson, PhD[3], Beverley Glass, PhD[1], Andrew J. Mallett, PhD[1,4,5]

[1]*College of Medicine & Dentistry, James Cook University, Townsville, QLD, Australia*
[2]*Department of Renal Medicine, Cairns Hospital, Cairns, QLD, Australia*
[3]*Sydney School of Public Health, The University of Sydney, Sydney NSW, Australia*
[4]*Department of Renal Medicine, Townsville University Hospital, Townsville, QLD, Australia*
[5]*Institute for Molecular Bioscience, The University of Queensland, Brisbane, QLD, Australia*

Correspondence: Tara Watters tara.watters@my.jcu.edu.au

Online Resource 4 – Supplementary Figures

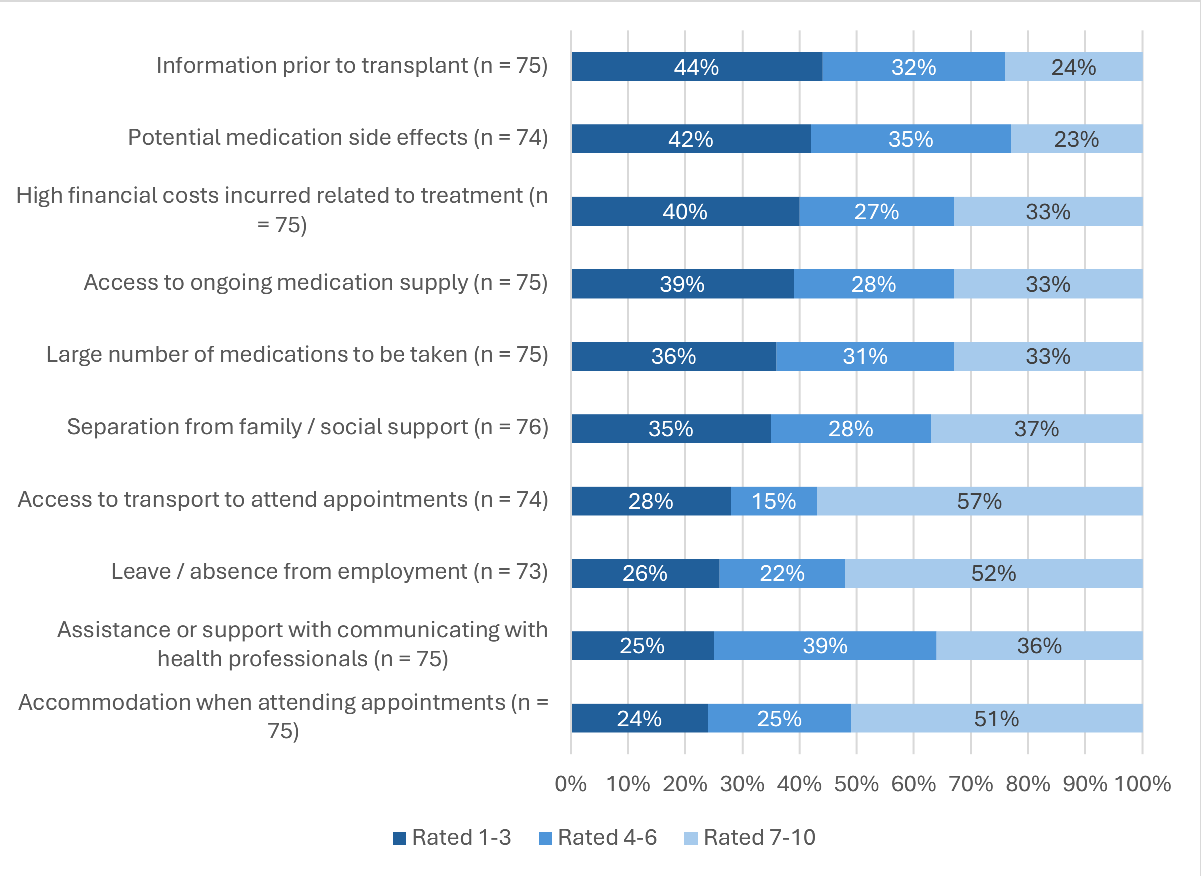

Supplementary Fig. 1 Importance of different aspects of the transplantation process to existing kidney transplant recipients. Responses from participants when asked to rate different kidney transplant aspects from 1-10 in order of importance, with 1 being most important and 10 being least important. Some participants did not rate all listed aspects, hence missing data excluded from analysis
