# Supplementary material for: Health professional and transplant recipient perspectives of kidney transplantation in regional, rural, and remote Australia – a survey study
Source: J Nephrol. 2025 Jun 16;38(5):1403–12. doi: 10.1007/s40620-025-02331-4 (PMC12289722; doi:10.1007/s40620-025-02331-4)
Supplement: Supplementary file 5 — Supplementary file5 (PDF 157 KB) [file 40620_2025_2331_MOESM5_ESM.pdf]

Health professional and transplant recipient perspectives of kidney transplantation in regional, rural, and remote Australia – A survey study

Journal of Nephrology

Tara Watters, BPharm (Hons)[1,2], Nicole Scholes-Robertson, PhD[3], Beverley Glass, PhD[1], Andrew J. Mallett, PhD[1,4,5]

[1]*College of Medicine & Dentistry, James Cook University, Townsville, QLD, Australia*
[2]*Department of Renal Medicine, Cairns Hospital, Cairns, QLD, Australia*
[3]*Sydney School of Public Health, The University of Sydney, Sydney NSW, Australia*
[4]*Department of Renal Medicine, Townsville University Hospital, Townsville, QLD, Australia*
[5]*Institute for Molecular Bioscience, The University of Queensland, Brisbane, QLD, Australia*

Correspondence: Tara Watters tara.watters@my.jcu.edu.au

Online Resource 5 – Supplementary Tables

Supplementary Table 1 – Themes and subthemes identified from free text responses and supporting participant quotations

**Themes**	**Quotes**
Enabling timely and flexible access to transplant assessment	
Ensuring earlier referral for transplant	"Earlier referral for transplantation assessment and workup." *(Neph; Reg)*<br><br>"Timely referral for eligibility assessment." *(Nur; Reg)*<br><br>"Improving the access to timely investigations and specialist involvement for rural and regional patients would improve their access to transplantation. In metropolitan centres patients are worked up from early in CKD journey compared to post commencement on dialysis in more regional centres and stalled by lack of access to specialist input." *(Neph; Reg)*
Increasing access to MDT input	"More pharmacist involvement in pre-transplant work up, currently doesn't exist in my area." (*Phar; Rem)*<br><br>"Social work input to support with logistics of travel and funding this." *(Diet; Reg)*<br><br>"Improved support services around weight loss and smoking cessation." *(Neph; Reg)*

Improved coordination of testing	“Trying to group together assessment investigations and review to prevent need for recurrent travel.” *(Neph; Metro)*<br><br>“Transplant centres need to assist rural patients by either bringing them into their hospital and completing all required tests in one visit or have navigators that patients can contact and then they facilitate the organisation of the tests. Also, some services in rural areas do not have the capability to do the required tests so the patient must choose to either do multiple trips for required tests or not proceed to transplantation.” *(Nur; Rur)*<br><br>“Simply the evaluation process by providing improved access to complex investigations such as cardiology assessments, angiogram, dental, psychological care.” *(Neph; Metro)*<br><br>“Rural patients take some time to get tests done (availability of cardiac testing, availability of dentistry) - having better availability for these may help although MOC for this tricky.” *(Neph; Reg)*<br><br>“When doing work up tests for live donor kidney transplant, be provided with all tests needing to be done at once rather than being drip fed them over several months.” *(Pat; Rem)*
Expanding outreach visits for transplant team	“Outreach clinics so that the members of the team from the transplant centre can see patients and their families/carers in their own environment. The transplant team get a much better understanding of the challenges faced by patients, and the patients and their support team get to build a rapport with the transplant team.” *(Neph; Metro)*<br><br>“Transplant team going to them especially for Aboriginal patients and this would include the entire team like social work and dietetics.” *(Soc; Metro)*<br><br>“Transplant assessment teams visiting regional areas.” *(Neph; Reg)*<br><br>“Reduced travel for work up and transplant team appointments.” *(Soc; Rur)*
Enhanced use of telehealth	“Telehealth assessment of patients by transplant team will reduce the pre-transplant work up time.” *(Neph; Rem)*<br><br>“Telehealth access for transplant assessment reviews.” *(Neph; Reg)*
Aligning communication and education with patient needs	
Increased and adaptable education provision	“Improve accessibility of patient education materials or delivery of education to patients and staff in regional rural and remote areas.” *(Nur; Metro)*<br><br>“When attending [transplant assessment] patients have a huge burden of information on that day often due to travel, would be good to spread that information out e.g., over 2 days to improve understanding.” *(Phar; Metro)*

	"Face to face education and follow up needs to be increased to ensure that informed decision making occurs." *(Nur; Reg)*<br><br>"If I had any knowledge about how sick I would get after receiving a kidney from a donor with CMV, I never would've accepted the kidney. More information should be available about the risks to patients who don't already have this virus." *(Pat; Reg)*<br><br>"More information regarding all side effects of all my medication." *(Pat; Reg)*<br><br>"Because of the other circumstances that I experienced in my earlier life that contributed to losing my kidney caused me trauma and to feel angry, which affected how I could engage with the doctors and treatment. There's no access to education about kidney treatments in the communities." *(Pat; Reg)*
Compassionate communication with clinicians and staff	"Better understanding from nursing staff. Our whole world gets turned upside down for the rest of our lives. We lose control of our life and so what control we have left should be respected." *(Pat; Reg)*<br><br>"More communication from nephrologists / transplant coordinators (RE: waiting lists, how you're feeling) and more info from transplanting hospital whilst on the list." *(Pat; Rur)*<br><br>"I was given incorrect information from the admin desk, and I ended up very, very upset thinking I had missed my opportunity." *(Pat; Rur)*
Reducing financial hardship	
Minimising out of pocket costs for travel and accommodation	"More flexible support from patient assistance transport schemes, currently the red tape and 'rules' they have means patients are not supported and common sense is lacking." *(Nur; Metro)*<br><br>"Increased financial support for rural patients who are required to travel and stay in capital cities for their transplantation and post-transplant care." *(Psyc; Reg)*<br><br>"Financial Support - waiving patient and escort travel and accommodation and supply transport or taxi vouchers." *(Soc; Reg)*<br><br>"Costs associated with living expenses post-transplant. While QLD Health subsidise accommodation you can't just choose the cheapest as they most often do not have vacancy. And cheapest accommodation is on average $100 per day more expensive." *(Pat; Reg)*<br><br>"Cost of travelling/accommodation to another state." *(Pat; Reg)*

Overcoming financial hardship	"Financial support for regional/rural/remote potential recipients is crucial. Especially the cost and time (for support person) involved in transplant operation during [metropolitan city] stay. Financial cost is one of the challenging barriers for many potential recipients in regional/rural/remote areas." *(Nur; Reg)*<br><br>"Money is always tight for me if I could have had the chance to save, but due to cost of living this was pretty much impossible. This made my time worrisome. I could not get compensation through work as I did not reach 3 months, so no work cover." *(Pat; Reg)*
Fostering comprehensive psychosocial support	
Embedding culturally appropriate support within transplant team	"Our Indigenous patients need Indigenous Liaison Officer and Indigenous Health Workers to be included in their 'transplant team' - to support them through their journey." *(Soc, Reg)*<br><br>"Involve First Nations Australian clinicians in the formal assessment discussion ("the committee" or whatever) & ensure there is both appropriate documentation and an avenue of appeal for both patients' wants and clinicians unhappy with the acceptance/non-acceptance decision." *(Neph; Metro)*<br><br>"Specialist surgeons need to understand the complexities of living in Aboriginal communities." *(Diet; Rem)*
Facilitating ongoing connection with existing support networks	"It was very hard to find a support person who would travel and be away from home for so long." *(Pat; Reg)*<br><br>"To have had the transplant somewhere a bit closer to home, so I could have been with my family." *(Pat; Rem)*<br><br>"Being closer to home to have easier access to family and friends which stimulates the mind when there is so much spare time in the weeks following the transplant." *(Pat; Reg)*
Developing health professional navigator roles	"More supports walking alongside patients to understand the process and working with patients in [regional area] (especially remote communities) to understand the barrier to transplant and how to overcome." *(Diet; Reg)*<br><br>"A nurse navigator type of person for these patients for transplant stuff will in my opinion make a huge impact." *(Neph; Reg)*<br><br>"As I had to travel a long distance and was on my own for arrival and the procedure, I would have liked to be able to meet a staff member from the transplant team to help me find my way." *(Pat; Rur)*
Engaging with peer support and mentoring	"Local mentorship and support group directly guided by a transplant team / physician." *(Neph; Metro)*<br><br>"Having someone to talk to who has already had a transplant would have been helpful just to understand what it was like for them and to be able to ask questions." *(Pat; Reg)*

	“Had mentoring from someone who had been through it as support and ongoing questions.” *(Pat; Reg)*<br><br>“A support group with other people who have been through a transplant as everyone's side effects etc. is different and we can compare notes. Talking to other people who understand where you are coming from where health professionals don't fully understand.” *(Pat; Reg)*
Accessing safe and appropriate accommodation	
“Accommodation that was suitable to transplant patients. More assistance in finding appropriate accommodation within walking distance to the hospital.” *(Pat; Reg)*<br><br>“An improvement in accommodation whilst in [metropolitan city] post-transplant. The accommodation I stayed in was like going back to the 70's. Being adequately accommodated is an important part of recovery.” *(Pat; Reg)*	
Advancing post-transplant provision of care	
Streamlining local access to medications	“Ensure patients have ongoing access to post-transplant medications to reduce the risk of avoidable negative clinical outcomes related to geographic location.” *(Phar; Reg)*<br><br>“Nationally standardise the post-op supply of medication, in [state] it is confusing that some medications are PBS listed from community pharmacy, some are HSD from hospital pharmacy and some are private prescriptions. For the average patient, managing all these is difficult, and their specialist who initially wrote the script is 200+km away, with no idea how hard this will be for them once they get back home.” *(Phar; Rur)*<br><br>“To access all medication at my local pharmacy, instead of having to go to the hospital.” *(Pat; Reg)*<br><br>“To be able to access my medications more easily.” *(Pat; Rem)*
Expediting turnaround of pathology results	“Local pathology for immunosuppressant levels. Currently there is a delay of 1-2 days to have a level return.” *(Phar; Reg)*<br><br>“Availability of the same day results/machine for tacrolimus level monitoring.” *(Neph; Reg)*<br><br>“Better access to laboratory testing and support for patients in rural/remote areas i.e., areas like [remote community].” *(Neph; Reg)*<br><br>“Better blood testing facilities in far north [state]. Tacrolimus results have to currently get flown to [metropolitan city] from [regional centre] causing a delay in results from anywhere between 24-72 hours. Not good in early stages of transplant.” *(Pat; Rem)*

Striving for provision of services closer to home	"The ability to be able to have regular monitoring from an external service that specialises in transplants. And/or the ability to have the transplant closer to home." *(Diet; Reg)*<br><br>"For these remote people to actually receive nephrology appointments on a regular basis be it telehealth or face to face." *(Nur; Reg)*<br><br>"Having to be close to the hospital for regular bloods, appointments with doctors/surgeons and scans etc." *(Pat; Reg)*<br><br>"The only downside was having to spend six weeks in [metropolitan city]. If we had a transplant unit in [regional centre] that part would have been much easier." *(Pat; Reg)*<br><br>"Closer to home, more professional guidance, post-transplant care." *(Pat; Reg)*
Sustaining ongoing management of medical and psychological needs	"I was suffering with pain for months after transplant, I was the only one while in [metropolitan city] with severe pain after 6 weeks." *(Pat; Reg)*<br><br>"I have developed anxiety / PTSD from years of medical procedures / surgeries. My cognitive function, memory, has become a problem." *(Pat; Reg)*<br><br>"More support with side effects of medication." *(Pat; Reg)*<br><br>"The boredom really got to me, made me feel depressed and frustrated, made me spend a lot of money." *(Pat; Rur)*<br><br>"I was very frightened when I heard about my kidney was fail. In experience I was lucky to have one of family member come with me while I receive my new kidney. I had to take in all the medication I had to take on a day-to-day basis." *(Pat; Rem)*<br><br>"Follow up - to see how patients are feeling and what to do going forward." (*Pat; Rur)*<br><br>"More psychological support available to transplant patients." *(Pat; Rur)*

Abbreviations: Neph – Nephrologist, Nur – Nursing Staff, Phar – Pharmacist, Diet – Dietitian, Soc – Social Worker or Indigenous Liaison Officer, Psyc – Psychologist, Pat – Patient, Metro – Metropolitan Area, Reg – Regional Area, Rur – Rural Area, Rem – Remote Area
