# Supplementary material for: Health professional and transplant recipient perspectives of kidney transplantation in regional, rural, and remote Australia – a survey study
Source: J Nephrol. 2025 Jun 16;38(5):1403–12. doi: 10.1007/s40620-025-02331-4 (PMC12289722; doi:10.1007/s40620-025-02331-4)
Supplement: Supplementary file 6 — Supplementary file6 (PDF 112 KB) [file 40620_2025_2331_MOESM6_ESM.pdf]

Health professional and transplant recipient perspectives of kidney transplantation in regional, rural, and remote Australia – A survey study

Journal of Nephrology

Tara Watters, BPharm (Hons)[1,2], Nicole Scholes-Robertson, PhD[3], Beverley Glass, PhD[1], Andrew J. Mallett, PhD[1,4,5]

[1]*College of Medicine & Dentistry, James Cook University, Townsville, QLD, Australia*
[2]*Department of Renal Medicine, Cairns Hospital, Cairns, QLD, Australia*
[3]*Sydney School of Public Health, The University of Sydney, Sydney NSW, Australia*
[4]*Department of Renal Medicine, Townsville University Hospital, Townsville, QLD, Australia*
[5]*Institute for Molecular Bioscience, The University of Queensland, Brisbane, QLD, Australia*

Correspondence: Tara Watters tara.watters@my.jcu.edu.au

Online Resource 6 – Supplementary References

S31. McGrath P, Holewa H. 'It's a regional thing': financial impact of renal transplantation on live donors. Research Support, Non-U.S. Gov't. *Rural Remote Health*. 2012;12:2144. doi:https://doi.org/10.22605/RRH2144

S32. Scholes-Robertson N, Blazek K, Tong A, et al. Financial toxicity experienced by rural Australian families with chronic kidney disease. *Nephrology (Carlton)*. Aug 2023;28(8):456-466. doi:10.1111/nep.14192

S33. Australian Goverment. *Supporting Living Organ Donors Program Guidelines*. 2021. https://www.health.gov.au/sites/default/files/documents/2021/03/supporting-living-organ-donors-program-guidelines.pdf. Accessed 20th Oct 2024

S34. Mathur AK, Xing J, Dickinson DM, et al. Return on investment for financial assistance for living kidney donors in the United States. *Clin Transplant*. Jul 2018;32(7):e13277. doi:10.1111/ctr.13277

S35. Crawford S, Germann R. “One day renal transplant workup program - a patient-centred, equity focused quality initiative” in *ANZSN Annual Scientific Meeting*,

Adelaide, Australia, 2024. [Online]. Available: https://anzsnasm.com/23593. Accessed 20th Oct 2024

S36. Lambooy S, Krishnasamy R, Pollock A, Hilder G, Gray NA. Telemedicine for Outpatient Care of Kidney Transplant and CKD Patients. Article. *Kidney Intl Rep*. 2021;6(5):1265-1272. doi:10.1016/j.ekir.2021.02.016

S37. Al Ammary F, Concepcion BP, Yadav A. The Scope of Telemedicine in Kidney Transplantation: Access and Outreach Services. *Adv Chronic Kidney Dis*. Nov 2021;28(6):542-547. doi:10.1053/j.ackd.2021.10.003

S38. Tang J, Howell M, Roger S, Wong G, Tong A. Perspectives of Kidney Transplant Recipients on eHealth: Semistructured Interviews. *Transplantation Direct*. 2022;8(12):e1404. doi:10.1097/txd.0000000000001404

S39. Huuskes BM, Scholes-Robertson N, Guha C, et al. Kidney transplant recipient perspectives on telehealth during the COVID-19 pandemic. *Transpl Int*. 2021;34(8):1517-1529. doi:https://doi.org/10.1111/tri.13934
